# CastorDB: a comprehensive knowledge base for *Ricinus communis*

**DOI:** 10.1186/1756-0500-4-356

**Published:** 2011-09-13

**Authors:** Shalabh Thakur, Sanjay Jha, Bharat B Chattoo

**Affiliations:** 1 Centre for Genome Research, Department of Microbiology and Biotechnology Centre, Faculty of Science, The M. S. University of Baroda, Vadodara-390002, India; 2 Department of Biotechnology, ASPEE College of Horticulture and Forestry, Navsari Agricultural University, Navsari, Gujarat-396450, India

## Abstract

**Background:**

*Ricinus communis *is an industrially important non-edible oil seed crop, native to tropical and subtropical regions of the world. Although, *R. communis *genome was assembled in 4X draft by JCVI, and is predicted to contain 31,221 proteins, the function of most of the genes remains to be elucidated. A large amount of information of different aspects of the biology of *R. communis *is available, but most of the data are scattered one not easily accessible. Therefore a comprehensive resource on Castor, Castor DB, is required to facilitate research on this important plant.

**Findings:**

CastorDB is a specialized and comprehensive database for the oil seed plant *R. communis*, integrating information from several diverse resources. CastorDB contains information on gene and protein sequences, gene expression and gene ontology annotation of protein sequences obtained from a variety of repositories, as primary data. In addition, computational analysis was used to predict cellular localization, domains, pathways, protein-protein interactions, sumoylation sites and biochemical properties and has been included as derived data. This database has an intuitive user interface that prompts the user to explore various possible information resources available on a given gene or a protein.

**Conclusion:**

CastorDB provides a user friendly comprehensive resource on castor with particular emphasis on its genome, transcriptome, and proteome and on protein domains, pathways, protein localization, presence of sumoylation sites, expression data and protein interacting partners.

## Introduction

*Ricinus communis *(*Euphorbiaceae *family) is an industrially important non-edible oil seed crop with several well established applications in industry. Castor bean genome is around 350 Mb and was sequenced and assembled in 4X draft by Chan *et al*. [1] using whole genome shortgun strategy and is predicted to contain 31,221 proteins, although the function of most of these proteins remains unknown. Thus, a comprehensive database has been developed to provide a useful resource by integrating information on genome, transcriptome, and proteome of *R. communis*. Sequence data of Castor bean plant was obtained from various resources like National Center for Biotechnology Information (NCBI) [2] and JCVI Castor Bean Genome Database [3]. Appropriate programs were developed to establish a connection with various databases for accessing the information using API. Important information extracted from the analyzed data was compiled in a back-end database using MySQL database server [4] for the construction of CastorDB. The information incorporated in CastorDB was generated by comparing the information extracted from different resources thus a comprehensive resource has been built for *R. communis *with information on protein domains, biosynthetic pathways, protein localization, and presence of sumoylation sites, gene expression data, and information on interaction between proteins. CastorDB not only provides researchers an opportunity to extract detailed biological information on any specific gene or protein from a single resource but also prompts the researcher to use the information to explore new information that is becoming available in plant genomics.

## Database Content

### Primary Data

#### Sequence Data

Sequence information on 31,221 proteins and genes of *R. communis *was downloaded from JCVI Castor Genome database [3] on January 12, 2009. Sequences from this database have unique locus identifiers, which were used during the analysis for distinguishing sequences from each other. A large number of sequences obtained were described as either hypothetical or predicted.

#### Expression Data

dbEST [5] is a division of NCBI that contains EST data and "single-pass" cDNA sequences from various organisms. About 60,000 ESTs from different tissues of *R. communis *were obtained from dbEST. Each EST sequence was mapped on genes by performing nucleotide BLAST [6] against mRNA sequences from *R. communis *with e-value cutoff 10^-6^.

#### GO Annotation

*R. communis *proteins were mapped with gene ontology information on the basis of GO annotation available for Pfam domains from Gene Ontology database [7,8]. The mapping of GO annotation to Pfam Domain was generated from data available from InterPro database for InterPro2GO mapping [9]. 11847 proteins were mapped with probable GO annotation in *R. communis*.

### Derived Data

#### Localization Data

**Figure 1:**
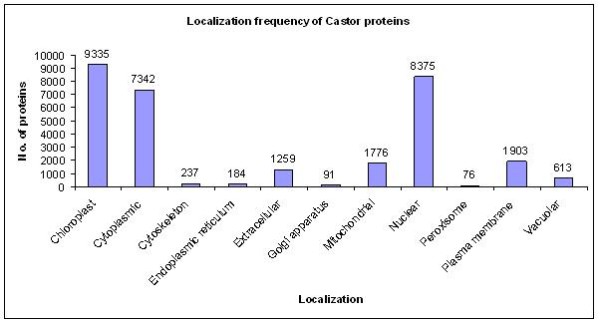
**Statistics of protein predicted at different cellular localization in *Ricinus communis***.

Prediction of the *R. communis *proteins localization was generated using the Wolf-PSORT [10], SignalP [11,12] and TMHMM [13,14] programs. WoLF-PSORT, which is a major extension to the PSORTII [15] program, predicts subcellular localization of proteins based on known sorting signal motifs and their amino acid sequences. SignalP 3.0 server predicts the presence and location of signal peptide cleavage sites in amino acid sequences from different organisms based on artificial neural networks and Hidden Markov Models. Integral membrane proteins in Castor bean genome were predicted by using TMHMM, which uses Hidden Markov Model to discriminate between soluble and membrane proteins. Frequency of proteins predicted at different cellular localization is shown in Figure 1.

#### Domains

**Figure 2:**
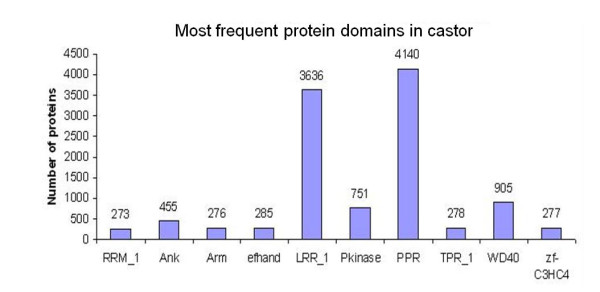
**Statistics of 10 high frequency domains predicted in *Ricinus communis***.

Pfam [16] database was used to predict domain present in *R. communis *protein sequences. Pfam, a large collection of multiple sequence alignments and Hidden Markov Models covering many common protein domains and families, has two parts; Pfam-A (a curated database with 9318 protein families) and Pfam-B, which contain large number of small families taken from PRODOM database [17] that do not overlap with Pfam-A. All *R. communis *protein sequences were scanned for probable domains using pfam_scan program with an E-value cut-off of 10^-3^. A total of 3546 domains were found for 18445 protein sequences, information for which is incorporated in CastorDB. Top 10 high frequency domains are shown in Figure 2.

#### Pathways

Putative pathways for the *R. communis *protein sequences were predicted by using KEGG Pathway database [18]. KEGG PATHWAY is a collection of manually drawn pathway maps representing knowledge on the molecular interaction and reaction networks and incorporating information for approximately 146,590 pathway maps from different species belonging to 407 reference pathways. *R. communis *proteins (31,221) were compared to the Swiss-Prot database [19,20] using BlastP [21] API from DDBJ [22] with an E-value cut-off of 10^-6^. Each query protein sequence from *R. communis *was assigned probable pathways based on pathway information available from KEGG database for their homologous protein sequences in other species. A total of 112 probable pathways were predicted for 3785 Castor bean proteins. All predicted pathways were manually checked to remove false positives from the prediction result.

#### Protein-Protein Interactions

**Figure 3:**
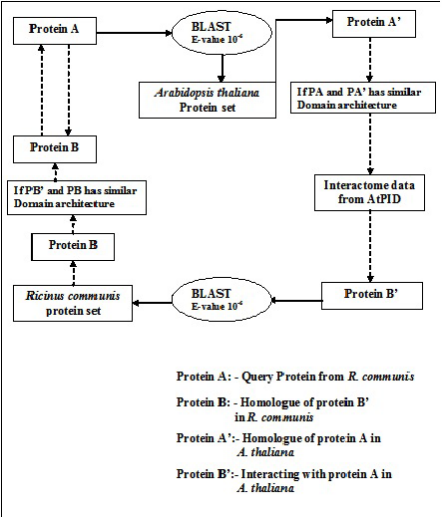
**Schematic representation showing algorithm for predicting protein-protein interaction in *Ricinus communis***.

Probable protein-protein interactions in *R. communis *were predicted using interaction information protein interaction for *Arabidopsis thaliana *from *Arabidopsis thaliana *Protein Interactome Database (AtPID) [23]. The AtPID represents a centralized platform to depict and integrate the information pertaining to protein-protein interaction networks, domain architecture, ortholog information and GO annotation in the *Arabidopsis thaliana *proteome. The Protein-protein interaction pairs in AtPID are predicted by integrating several methods with the Naive Baysian Classifier. Proteins from *R. communis *were BLAST against the *Arabidopsis thaliana *protein sequences obtained from The Arabidopsis Information Resource (TAIR) [24] and vice versa using E-value cutoff 10^-6^. The *R. communis *proteins which were predicted to show similar domain architecture (i.e. same domains) to that of homologue proteins from *A. thaliana *were only selected for further predicting probable interacting protein pairs. A total of 33,000 interacting protein pairs were predicted during the analysis. Schematic diagram showing prediction of protein-protein interaction in *R. communis *is shown in Figure 3.

#### Sumoylation sites

Putative sumoylation sites in *Ricinus communis *proteins were predicted using SUMOsp 2.0 [25] software for sumoylation site prediction by the Cuckoo work group. The non-redundant training data in software contained 279 sumoylation sites from 166 distinct proteins. SUMOsp 2.0 predicted sumoylation sites for 9755 protein sequences in *R. communis *at a high cut-off value.

#### Biochemical properties

Biochemical properties of the protein sequences were calculated using Pepstats program from European Molecular Biology Open Software Suite (EMBOSS) package [26]. Pepstats was programmatically linked and used to predict biochemical properties of *R. communis *proteins. Pepstats calculated molecular weight, isoelectric point, charge, size of protein, extinction coefficient and average residue weight for all the proteins in *R. communis*.

#### Best NCBI and KEGG Homologue

In order to find the best homologue for *R. communis *protein sequence in NCBI [2] and KEGG [18], protein BLAST [21] was performed at e-value cutoff 10^-10 ^against protein sequence dataset obtained from NCBI and KEGG using keyword *Ricinus communis*. The hit with maximum identity and lowest e-value was selected as best homologue.

### Architecture and Design of CastorDB

**Figure 4:**
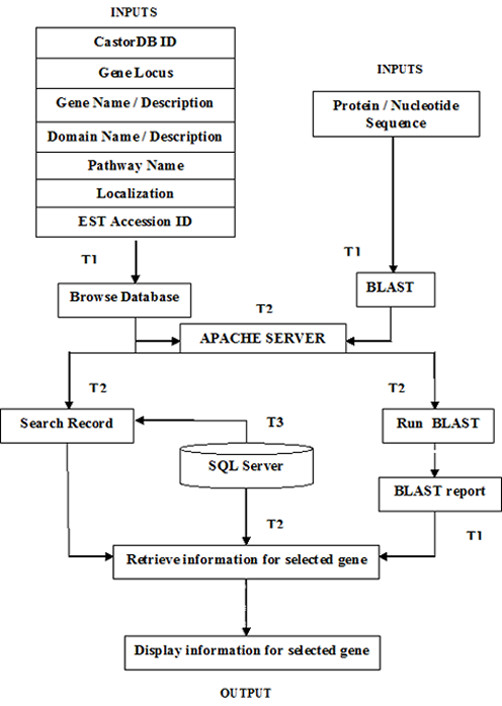
**Schematic representation of architecture of CastorDB showing different input options**. T1, T2 and T3 represent the members of Tier1, Tier 2 and Tier 3.

The architecture and design of CastorDB (Figure 4) consists of three tiers (T). T1: User Interface developed using HTML and Javascript [27], T2: Programs and Perl CGI [28] scripts for analysis, T3: MySQL Database storing raw data. T2 connects T1 with T3 and also supports use of analytical program like BLAST for sequence based analysis. The CGI scripts in T2 retrieve information from T3 and represent it on browser. The represented information again links to scripts and program in T2 and also provides links to various resources for further information.

#### Tier 1: User Interface

Graphical interface provides the user access to CastorDB using various input queries and provide links to additional information pages which guide the user during browsing of CastorDB. The query inputs from user interface are sent to program and scripts in layer T2 via post method.

#### Tier 2: Programs for analysis

T2 consists of Apache web server [29] for Windows platform and scripts written using Perl CGI [28]. Perl CGI scripts use bioperl modules to support use of local BLAST [21] obtained from NCBI ftp site and parse result to represent the necessary information on browser. CGI scripts also use MySQL Perl API to connect to the MySQL database [4] in tier T3. Perl DBI module along with DataBase Driver (DBD) for different type of server provides a generic interface for database access. Complex queries that analyze a large variety of different types of data can, therefore, be realized in a fairly intuitive manner.

#### Tier 3: Database Schema

The Relational Database Management System MySQL [4] was used to store data integrated in CastorDB. MySQL run as a server and provides multiple-user access to number of different databases. The database schema had been implemented using MySQL Perl API, an Application Programming Interface (API), for accessing data in a heterogeneous environment of relational and non-relational database management systems in Perl programming language.

### Data Retrieval

#### Web Interface Access

CastorDB provides access to explore the stored information by three different kinds of search methods: (i) Simple Search (ii) Advanced Search (iii) BLAST Search using protein or nucleotide sequence.

#### Simple Search

**Figure 5:**
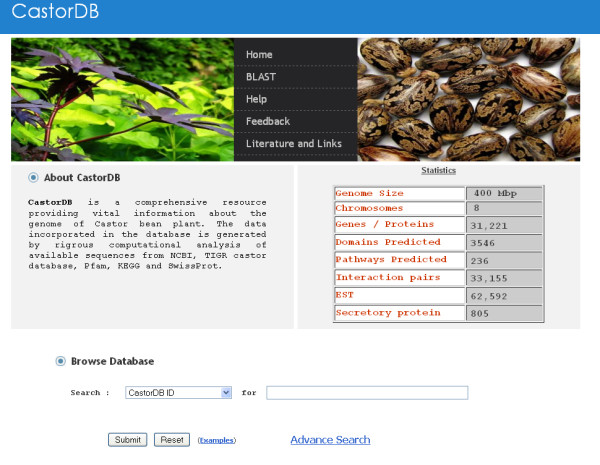
**Screenshot of the web interface for simple search of CastorDB using different query options**.

This feature of CastorDB allows user to browse database by inputting keyword for selected query option. There are seven query options (Figure 5) which accept specific input for retrieval of corresponding information from the database. Each gene/protein record in Castor DB is assigned a unique nine letter accession code termed as CastorID which begin with keyword "RC" and is followed by seven digit number (RC00#####). This ID differentiates each entry in the database from one another.

#### Advanced Search

**Figure 6:**
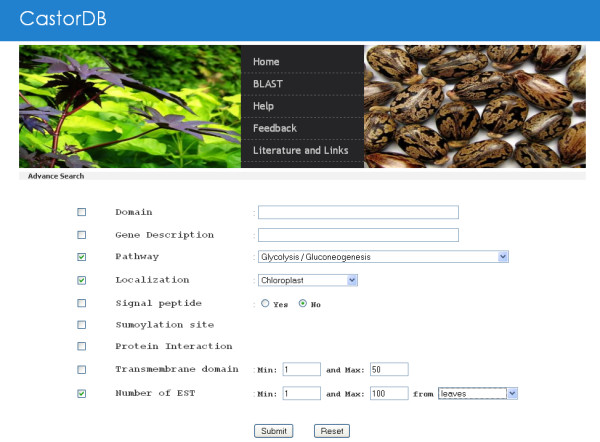
**Screenshot of the advance search interface of CastorDB, combining different query options**.

This mode of searching CastorDB allows user to combine multiple queries with one another. Database can be searched in multiple dimensions looking for records which satisfy the given conditions for all combined queries. For example: Query can be generated to search for genes having at EST's from leaves, involved in glycolysis pathway and localized in chloroplast of cell. Similarly many other queries can be generated using available options (Figure 6).

#### BLAST Search

BLAST [6,21] based search allows user to browse CastorDB using sequence in FASTA format. The option allows search against protein and nucleotide sequence database of *R. communis *generated using formatdb from standalone BLAST package. The result table generated after running the program display BLAST hits sorted according to percent identity in descending order.

### Representation of analysis results

**Figure 7:**
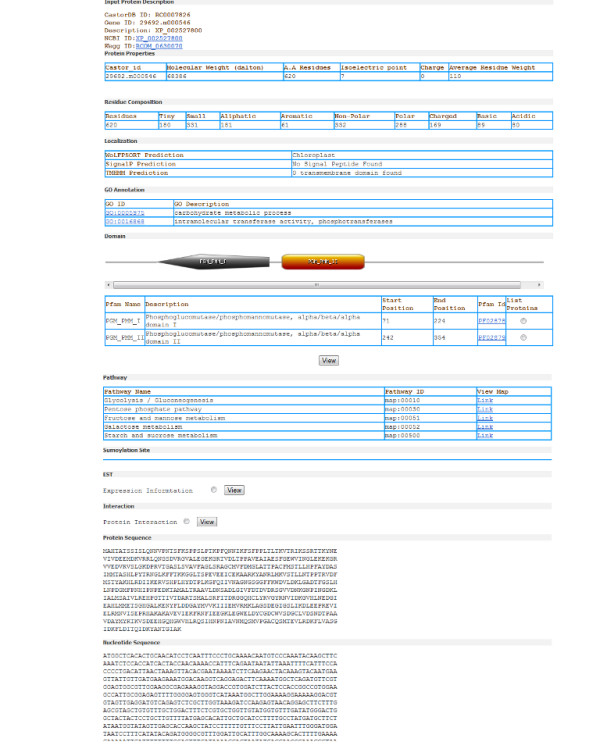
**Screenshot of the result page showing different information incorporated in CastorDB**.

**Figure 8:**
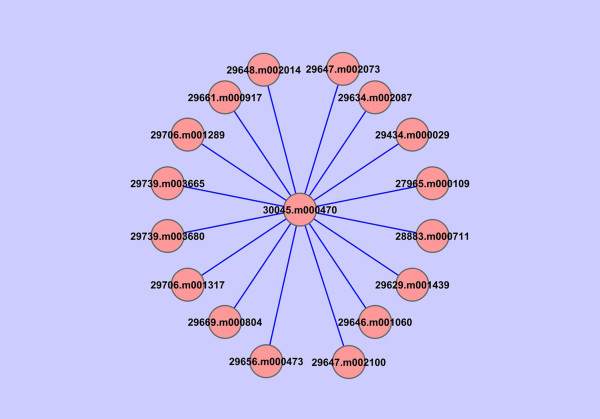
**Protein-protein interaction network visualized using Cytoscape**.

Information section for selected gene provides information about Domains along with image generated using Domain Image Generator program from Prosite [30], Pathways, Localization, Sumoylation site, EST expression, Protein-Protein interactions, biochemical properties and closest NCBI homologue (Figure 7). The graphical interaction network for selected protein can be visualized using Cytoscape software [31]. The link is provided to download "jnlp" file for each protein which run Cytoscape program using java web start (Figure 8).

### Exporting data

This feature allows user to download information in form of text file for all gene appearing in search result using multiple export option or by selecting each gene individually.

### Other web interfaces

Other web interfaces includes "Help" section which provides description of each query option and accepted keyword input in CastorDB. "Literature and Links" section provides links to external literature databases such as Pubmed and Agricol; and links to web resources used during analysis of castor genome. "Feedback" section allows user to comment on data and utilities incorporated in CastorDB.

## Discussion

The queries provided by CastorDB are focused on retrieving available information from various databases along with queried information for a particular gene or protein in *R. communis*. Currently, information about this important oil seed plant is available in different sources. Among the existing databases, (i) JCVI Castor Genome Database and NCBI provides sequence information on *R. communis *genes and proteins; (ii) Information on EST expressed during different condition is available from dbEST division of NCBI database.

CastorDB is, designed to facilitate the analysis of information on *R. communis *obtained from various resources and develop a comprehensive database. CastorDB database provides researchers information not only on gene and protein sequences but also on possible Go annotation, domains present in a protein, predicted pathways, probable interacting partners, sub-cellular localization, protein sumoylation sites, gene expression and even biochemical properties of a given protein. In addition to a common BLAST search, CastorDB provides the user with a scope for keyword search using the options like CastorDB ID, locus tag, gene name, domain name, pathway, localization, EST accession number. Also, some of the experimental data obtained from external resources are represented in more interpretable form which can provide researchers with a better understanding about the plant and help in designing critical experiments to gain deep insights into its biology. In order to incorporate newer findings the database will be updated in every 6 months.

## Conclusions

CastorDB was generated by correlating the information available on its genome, transcriptome, and proteome and a comprehensive resource was built on protein domains, pathways, protein localization, presence of sumoylation sites, expression data, protein interacting partners, *etc*. In addition to a common BLAST search and simple keyword search, CastorDB provides the user with a scope of doing advanced search by using different keywords and options. Also, some of the experimental data obtained from external resources are represented in more interpretable form. Thus, CastorDB would be an important database providing researchers with information to better understand the biology of this important plant.

## Availability and requirements

Project Name: CastorDB: a comprehensive knowledge base for *Ricinus communis*

Project homepage: The database is currently available at http://CastorDB.msubiotech.ac.in

Operating system(s): Platform independent

Programming language(s): HTML, Perl, CGI, Java, Javascript

License: Free for academics, Authorization is needed for commercial use (Please contact the corresponding author for more details)

## Competing interests

The authors declare that they have no competing interests.

## Authors' contributions

ST developed programs, scripts, tools for the database, carried out data analysis and drafted the manuscript; SJ helped in conceiving and designing the web server idea, analyzing the data wrote the manuscript; BBC provided critical inputs to develop the database, and to write the manuscript. All authors have read and approved the final manuscript.
